# Multiple source genes of HAmo SINE actively expanded and ongoing retroposition in cyprinid genomes relying on its partner LINE

**DOI:** 10.1186/1471-2148-10-115

**Published:** 2010-04-29

**Authors:** Chaobo Tong, Xiaoni Gan, Shunping He

**Affiliations:** 1 Laboratory of Fish Phylogenetics and Biogeography, Institute of Hydrobiology, Chinese Academy of Sciences, Wuhan, Hubei 430072, China; 2 Graduate School of the Chinese Academy of Sciences, Beijing 100039, China

## Abstract

**Background:**

We recently characterized HAmo SINE and its partner LINE in silver carp and bighead carp based on hybridization capture of repetitive elements from digested genomic DNA in solution using a bead-probe [1]. To reveal the distribution and evolutionary history of SINEs and LINEs in cyprinid genomes, we performed a multi-species search for HAmo SINE and its partner LINE using the bead-probe capture and internal-primer-SINE polymerase chain reaction (PCR) techniques.

**Results:**

Sixty-seven full-size and 125 internal-SINE sequences (as well as 34 full-size and 9 internal sequences previously reported in bighead carp and silver carp) from 17 species of the family Cyprinidae were aligned as well as 14 new isolated HAmoL2 sequences. Four subfamilies (type I, II, III and IV), which were divided based on diagnostic nucleotides in the tRNA-unrelated region, expanded preferentially within a certain lineage or within the whole family of Cyprinidae as multiple active source genes. The copy numbers of HAmo SINEs were estimated to vary from 10^4 ^to 10^6 ^in cyprinid genomes by quantitative RT-PCR. Over one hundred type IV members were identified and characterized in the primitive cyprinid Danio rerio genome but only tens of sequences were found to be similar with type I, II and III since the type IV was the oldest subfamily and its members dispersed in almost all investigated cyprinid fishes. For determining the taxonomic distribution of HAmo SINE, inter-primer SINE PCR was conducted in other non-cyprinid fishes, the results shows that HAmo SINE- related sequences may disperse in other families of order Cypriniforms but absent in other orders of bony fishes: Siluriformes, Polypteriformes, Lepidosteiformes, Acipenseriformes and Osteoglossiforms.

**Conclusions:**

Depending on HAmo LINE2, multiple source genes (subfamilies) of HAmo SINE actively expanded and underwent retroposition in a certain lineage or within the whole family of Cyprinidae. From this perspective, HAmo SINE should provide useful phylogenetic makers for future analyses of the evolutionary relationships among species in the family Cyprinidae.

## Background

Retrotransposons are widely distributed among eukaryotic genomes and occupy a substantial fraction of genome. These repeats increase in number by retroposition, which involves transcription of their genomic copies followed by reverse transcription of an RNA intermediate and results in cDNAs reintegration into the genome host [2-4]. Retrotransposons are divided into LTR elements, long interspersed nuclear elements (LINEs), and short interspersed nuclear elements (SINEs)[5].

Both LINEs and SINEs become amplified in genomes during evolution, but while LINEs encode the enzymes required for their retrotransposition, SINEs presumably borrow these enzymes from other sources, most likely from LINEs [6,7]. Researchers have observed that the sequences of many SINE and LINE pairs isolated from many organisms are similar in their 3'end regions [8-11]. Recently, we characterized a couple of SINE and LINE, designated HAmo SINE and its partner LINE, in silver carp and bighead carp based on hybridization capture of repetitive elements from digested genomic DNA in solution using a bead-probe [1]. We found that HAmo SINEs was active and amplified recently in the genomes of these two young species utilizing the enzymatic machinery for retroposition of HAmoL2 [1].

SINEs are short (approximately 80 to 400 bp) repetitive elements that often are present at more than 10^5 ^copies per genome. Many SINEs can be categorized into families based on sequence similarity and into subfamilies based on the presence of diagnostic nucleotides and/or deletions. An active member of a subfamily yields new copies through retroposition. After their dispersion within the genome, individual nonactive members of a subfamily accumulate mutations randomly. Thus, the average sequence divergence of members of a subfamily can be used to roughly estimate the age of the subfamily [12].

A typical SINE is composed of three parts: the tRNA-related region, the tRNA-unrelated region and the LINE-derived region with a polyA or short duplication tail. The existence of RNA Pol III promoters in the tRNA-related region ensures the transcriptional activity of SINEs, whereas the LINE-derived region allows the SINE to utilize the LINEs's enzymes for retrotransposition. The central region (the tRNA-unrelated region) between the tRNA- and LINE-derived regions is quite different and is not strongly conserved between different families, although two conserved central regions have been reported for two SINE families (V-SINE and Core-SINE) [13,14].

To reveal the species distribution and evolutionary history of SINEs and LINEs in more cyprinid genomes, we performed a multi-species search for HAmo SINE and its partner LINE in almost all subfamilies of Cyprinidae, as well as in distantly related fishes (including other four families of order Cypriniforms and the other orders Siluriformes, Polypteriformes, Lepidosteiformes, Acipenseriformes, Osteoglossiforms) using the bead-probe capture and internal-SINE-primer polymerase chain reaction (PCR) techniques. The detailed sequence alignment and nucleotide diversity estimation of HAmo SINE characterized to date enabled us to understand amplification and evolution of HAmo SINE in cyprinid fishes.

## Methods

### Tissue and DNA samples

Table 1 lists the fish species examined in this study and their geographic sources. Chen et al.'s latest taxonomic revision divides the family Cyprinidae into 12 subfamilies: Danioninae, Leuciscinae, Cultrinae, Xenocyprinae, Hypophthalmichthyinae, Cobioninae, Gobiobotinae, Acheilognathinae, Barbinae, Labeoninae, Schizothoracinae, and Cyprininae [15]. According to the recent cyprinid molecular phylogeny based on the rag2 gene, monophyly for the subfamilies Cyprininae and Leuciscinae and for the tribes Labeonini, Gobionini, Acheilognathini, and Leuciscini was well resolved with high nodal support. However, the cyprinid taxa endemic in East Asia emerged as a young monophyletic clade referred to as Xenocypridini. In this study, we selected 12 typical species to represent all subfamilies of family Cyprinidae as well as 5 other conflicting species that belong to the Xenocypridini (Table 1). All species DNA was isolated from ethanol-fixed tissues (fins or muscle) by incubation with proteinase K followed by phenol/chloroform extraction [16].

**Table 1:** Species analyzed and their geographic sources

*Subfamily*	*Taxa*	*Sampling location*	Ra2 gene
Hypophthalmichthyinae	*Hypophthalmichthys molitrix*	Wuhan, Hubei Prov.	DQ367002
	*Aristichthys nobilis*	Wuhan, Hubei Prov.	DQ367038
Xenocyprinae	*Distoechodon hupeinensis*	Jingkou, Hubei Prov.	DQ366998 (a)
	*Xenocypris argentea*	Taoyuan, Hunan Prov.	DQ367024
Cultrinae	*Culter alburnus*	Taoyuan, Hunan Prov.	DQ367004
Leuciscinae	*Leuciscus tumensis*	Tumen, Jilin Prov.	This paper
	*Ctenopharyngodon idella*	Jingkou, Hubei Prov.	DQ366996
	*Squaliobarbus curriculus*	Taoyuan, Hunan Prov.	DQ367021
	*Elopichthys bambusa*	Taoyuan, Hunan Prov.	DQ367016
	*Mylopharyngodon piceus*	Jingzhou, Hunan Prov.	DQ367011
Acheilognathinae	*Rhodeus ocellatus*	Wuhan, Hubei Prov.	This paper
Gobioninae	*Saurogobio dabryi*	Wangzhou, Chongqing.	DQ367020
Cyprininae	*Cyprinus carpio*	Wuhan, Hubei Prov.	DQ366994
Labeoninae	*Garra orientalis*	Ledong, Hainan Prov.	DQ366957
Schizothoracinae	*Shizothorax grahami*	Kuming, Yunnan Prov	DQ366989 (b)
Barbinae	*Barbodes opisthoptera*	Mengla, Yunnan Prov.	DQ366952 (c)
Danioninae	*Opsariichthys bidens*	Taoyuan, Hunan Prov.	DQ367014
	*Danio apogon*	Mengla, Yunnan Prov	DQ367039
	*Danio rerio*	GenBank	NC_007136
Outgroup	*Myxocyprinus asiaticus*	Wuhan, Hubei Prov	DQ367043
	*Micronemacheilus pulcher*	Rong'an, Gaungxi Prov.	DQ367041

The rag2 sequences of the noted (a b c) three species was substituted by another species in the same genus, because of the absence of their rag2 sequence in ncbi. A: Distoechodon tumirostris B: Schizothorax meridionalis C: Barbodes huangchuchieni

### Isolation of SINE and LINE sequences using bead-probe capture and internal-primer-SINE PCR techniques

A rapid bead-probe capture technique based on magnetic separation was used to isolate full-size of HAmo SINE copies from unknown genomes as described previously [1]. The clone Hmo41_It was bound to beads to act as the probe for capturing HAmo SINE similar sequences from a HaeIII-fragmented genomic DNA pool (Table 1). The beads were washed once with 200 μL hybridization buffer (5 × SSC, 0.1% SDS) for 5 min, then 150 μL of the denatured genomic pool were added to the resuspend the beads. Hybridization at 55°C took place for 2 hours, then non-complementary sequences were removed by washing successively with 400 μl TEN1000 (10 mM Tris-HCl, 1 mM EDTA, 1000 mM NaCl, pH 7.5) three times for 5 min each; and 400 μl buffer (0.2×SSC,0.1%SDS) three times for 5 min; 400 μl TEN1000 for 10 min. Finally, the captured fragments were amplified by PCR and cloned directly into T-vector for sequencing. Additionally, many LINE sequences were isolated simultaneously during the above process because the probe contains the common tail shared by SINE and LINE.

Internal-primer-SINE PCR was conducted to amplify the internal region corresponding to residues 18-144 of the whole HAmo SINE consensus sequences (residues 1-150) using a pair of primers (primer ItF, 5-TGGTTAGAGCATGGCACTAGCAA-3, primer ItR, 5-TGCATTTGGCAGACGCTTTTATC-3). The PCR was run in a total volume of 20 μl including 200 ng DNA template with 25 cycles of 95°C for 40 s, 62°C for 40 s, and 72°C for 40 s. The internal-primer-SINE-PCR was conducted in the selected 17 species of Cyprinidae to detect many individual SINE copies. Finally, about 10 clones were characterized and sequenced for each species.

### Naming of Clones

Many HAmo sequences (full-size or internal) were isolated and characterized from distinct cyprinids fishes. Each locus was named after the number of the clone and the name of the species from which it was isolated. In the middle, F or I was used to represent the internal SINE sequence or full-size sequence obtained from the bead-probe capture (F) or internal-primer-SINE PCR (I), respectively. For example, Ltu_I_4 means that the number of the clone was 4 and it was isolated from *Leuciscus tumensis *based on the internal-primer-SINE PCR.

### Estimation of copy number using quantitative RT-PCR

Plasmid Hmo41_It and Genomic DNA were prepared as the standard and samples for Real-Time PCR, respectively (Table 2). Their concentrations then were measured using a spectrophotometer, and serial dilutions were prepared as templates to perform RT-PCR in a PCR machine (Bio-Rad, Chromo4 HERCULES, CA, USA) together. All Real-Time PCR was performed with 40 cycles at 95°C 40 s, 62°C 40 s, 72°C 40 s including Primer ItF and ItR (300 nM final concentration) and SYBR GREEN in a final volume of 25 uL. At last, a melting curve analysis was done after the amplification phase. The standard curve and data analysis were carried out in the software MJ Opticon Monitor 3.1.

**Table 2:** Copy number estimation of SINE by quantitative RT-PCR

Standard	Serial Con	CT	Cultivation:	
Plasmid (Hmo_41)	0.2 μg/μl	11.10	Y = -0.2989x+14.22	
	0.02 μg/μl	16.30	Y = -0.2989x+14.22	
	0.002 μg/μl	18.36	R ^2 = 0.997; E = 0.99	
	0.0002 μg/μl	21.72	Results: plasimid DNA size = 2× 103 bp copy number of plasmid per μl = 6.5× 10^11 ^(2 ug/ul)	
	0.00002 μg/μl	25.30		
**Sample**	**Con/(ug/ul)**	**CT**	**PCNH**	**Avg.**
Xenocypris argentea	0.115	13.32	183221.1127	3.0 × 10^5^
	0.0115	15.44	425899.8786	
Cutler alburnus	0.9	12.12	53470.08164	3.5× 10^4^
	0.09	17.11	17242.48462	
Elopichthys bambusa	0.133	15.32	39996.47484	4.0 × 10^4^
	0.0133	18.69	39330.43191	
Squaliobarbus curriculus (Genome size≈ 1 pg)	0.1	13.83	148331.8306	1.7 × 10^5^
	0.01	16.8	192088.4962	
Mylopharyngodon piceus (Genome size≈ 1 pg)	0.36	15.04	17916.89943	1.7× 10^4^
	0.036	18.46	17022.5593	
Ctenopharyngodon idellus (Genome size≈ 1 pg)	0.314	15.32	16941.18202	1.7 × 10^4^
	0.0314	18.69	16659.06829	
Leuciscus tumensis	0.104	13.32	202600.2689	3.3× 10^5^
	0.0104	15.44	470946.9811	
Saurogobio dabryi	0.03	11.91	1853536.326	1.7× 10^6^
	0.003	15.51	1555826.276	
Rhodeus ocellatus (Genome size≈ 1 pg)	0.092	13.91	152592.9712	2.2× 10^5^
	0.0092	16.23	309091.1208	
Cyprinus carpio (Genome size≈ 2 pg)	0.0033	22.06	31174.78108	3.1× 10^4^
	0.00033	25.39	31511.30668	
Shizothorax grahami	0.08	10.66	3286149.864	5.1× 10^6^
	0.008	12.87	7179899.5	

Con: concentration; CT: (cycle threshold) is defined as the number of cycles required for the fluorescent signal to cross the threshold; PCNH: Predicted copy no. in haploid genome; SC: standard curve; The R^2 ^value is the coefficient that is used to assess the fit of the standard curve to the data points plotted. The R2 value was > 0.99 which is the required value for reliable quantitation. The efficiency of the PCR reaction (E) is calculated using the formula E = (10^(-1/slope)^- 1), where the slope is calculated from a standard curve plot of Ct values against the logarithm of template amount. A value close to 1 indicates high PCR efficiency. PCNH was calculated using the equation: PCNH = Genome size (pg)/Genomic DNA Con (μg/μl) × 10^Y^/10 ^21.72^× 6.5× 10^7^. The genome size information came from http://www.genomesize.com/.

### Construction of phylogenetic relationships of 17 fish species based on the rag2 gene

Phylogenetic analysis was performed using Bayesian inference within the program MrBayes 3.1.1 [17,18]. The rag2 sequences used in this study were reported previously by our group [19]. For the Bayesian analysis, two million generations were completed, with tree and parameter values recorded every 1000 generations. Four chains were run in each of the two independent analyses that MrBayes executed as a default. The chain heating temperature was set to 0.2. At the end of the analysis the first 25% of stored trees were eliminated by setting sumtburnin to 1000, and the remaining trees were automatically compiled into a consensus tree by the program.

## Results

### Multi-species searching for HAmo SINE within the Cyprinidae

**Figure 1:**
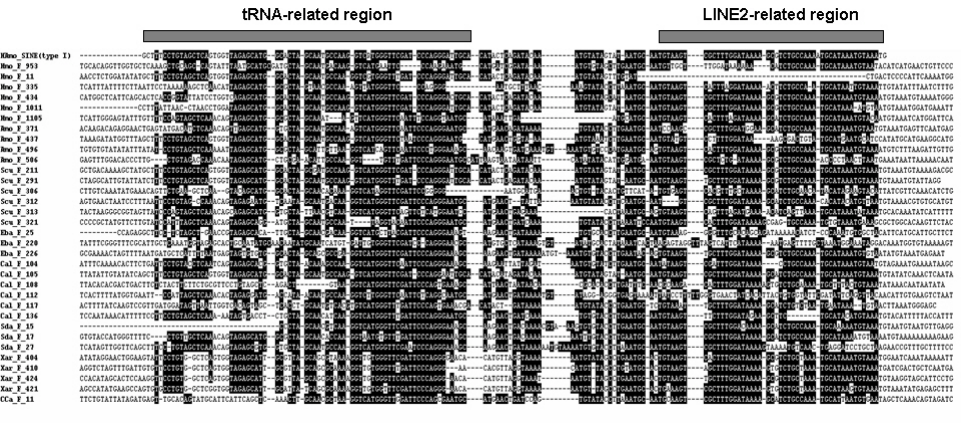
**Alignment of 34 new HAmo members isolated based on the bead-probe capture from eight species**. Hmo: *Hypophthalmichthys molitrix; *Amo: *Aristichthys nobilis; *Scu: *Squaliobarbus curriculus*; Eba: *Elopichthys bambusa*; Cal: *Cutler alburnus*; Sda: *Saurogobio dabryi*; Xar: *Xenocypris argentea; *Cca: *Cyprinus carpio*. 3 members (Scu_F_270, Scu_F_156, Sda_F_9) are not shown because they have too long insertion in the central region. The top of alignment is the consensus sequence which is identical to the HAmo SINE consensus sequence in silver carp and bighead carp. The tRNA-related region and LINE2-derived region are shown by thick bar. Dashes indicate gaps inserted to improve the alignment. Nucleotides at the position over 55% of similarity are highlighted. The GenBank accession numbers of them are as follows: GQ370825-GQ370860.

Figure 1 shows the alignment of 37 novel full-size SINE sequences isolated and characterized based on the magnetic separation system. These SINEs were similar to the consensus sequence of HAmo SINE reported for silver carp and bighead carp, which has a tRNA^lys^-related promoter region at the 5'end, a unique family-specific region, and an end with a HAmo-LINE2-derived 3' terminus preceding the TAAATG short tandem repeat. Remarkably, HAmo SINE seems to be widely distributed in cyprinid genomes and multiple subfamilies may exist based on preliminary observation of their high diversity.

Internal-primer-SINE PCR which amplified fragments corresponding to residues 18-144 of the HAmo SINE consensus sequence (residues 1-150) were conducted. The result showed that HAmo SINE was present in all 17 representatives of the family Cyprinidae tested in this study. To unambiguously confirm them and obtain enough HAmo SINE sequences to observe their diversities, we cloned the positive fragments and sequenced about 10 positive clones for each species. Moreover, 125 internal sequences corresponding to different individual HAmo-SINE copies from the 17 cyprinid genomes were isolated and characterized.

### Four subfamilies and their different patterns of polyA insertion and short tandem duplication in tRNA-unrelated region

**Figure 2:**
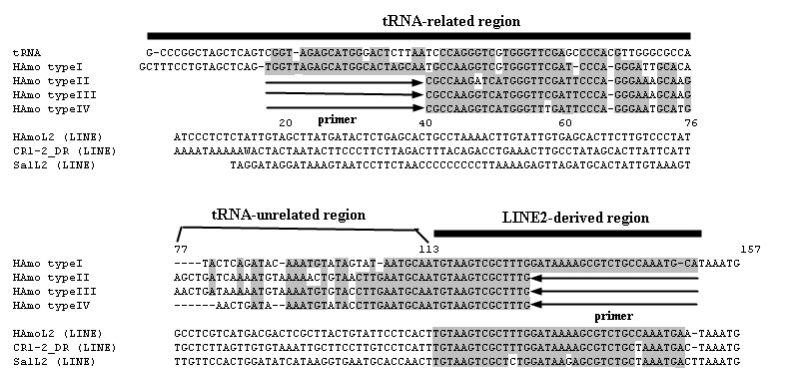
**Comparison of consensus sequences of four HAmo SINE subfamilies, type I, II, III and IV**. The tRNA-derived region of three SINE families and the tRNA^lys ^(CUU) in rabbit are aligned. The different family-specific regions represented the most diagnostic nucleotides. The tail regions of these SINEs and their partner LINEs tail are compared. When most of nucleotides at a given position are identical, they are shaded. The GenBank accession numbers of novel HAmo LINE2 sequences are as follows: GQ370975-GQ370988.

Sixty-seven full-size sequences and 125 internal-SINE sequences (plus 34 full-size and 9 internal sequences previously reported in bighead carp and silver carp) [1] from 17 species of the family Cyprinidae were aligned. On the basis of the presence of the diagnostic nucleotides, four subfamilies (type I, II, III and IV) exist, each of which is supported by abundant sequences from many distantly related species. Figure 2 shows the alignment of the consensus sequences of the four HAmo SINE subfamilies; the consistent nucleotide changes for each subfamily (diagnostic nucleotides) were recognized at 30 positions. Most of the diagnostic nucleotides (23 of 30) were distributed in the tRNA-unrelated region of HAmo SINE, and the diagnostic nucleotides were composed of different patterns of polyA insertion, short tandem repeats, and deletion or mutation in the respective subfamilies.

**Figure 3:**
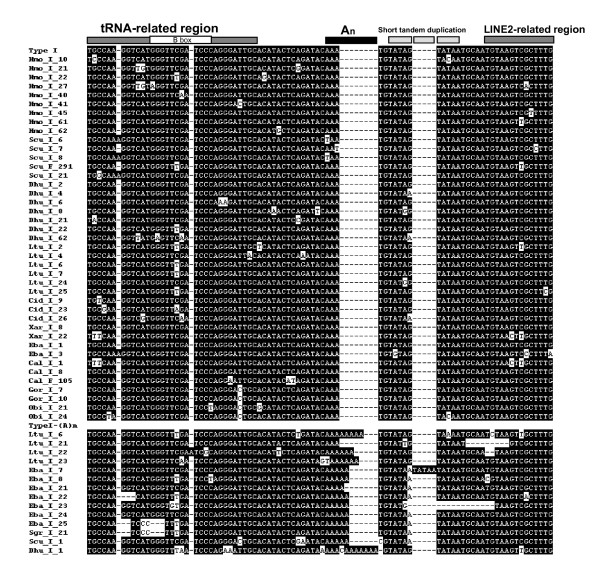
**Alignment of HAmo Type I members**. The consensus sequences are shown on top. The most frequently occurring nucleotide at a position was chosen as the consensus nucleotide at the position. The tRNA-derived region and LINE-related region of SINE are shown by thick bar. In the central region, there are a variable polyA(1-14) and TATAA repeated 2-3 times. The GenBank accession numbers of them are as follows: GQ370861-GQ370903.

**Figure 4:**
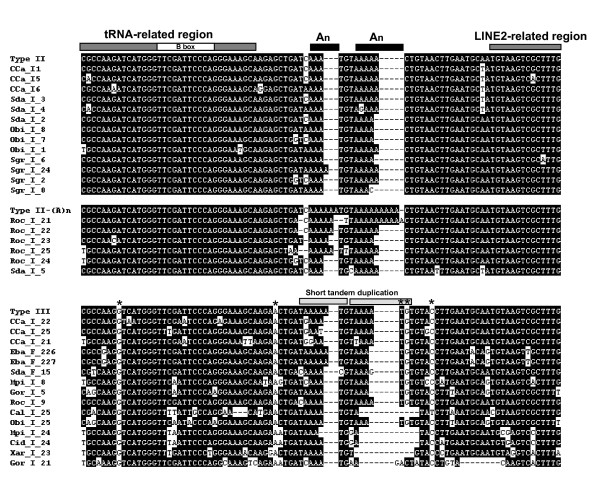
**Alignment of HAmo Type II and Type III members**. The consensus sequences are shown on top. The most frequently occurring nucleotide at a position was chosen as the consensus nucleotide at the position. The tRNA-derived region and LINE-related region of SINE are shown by thick bar. Star indicates the diagnostic nucleotide between type II and type III. In the central region, type II has two variable polyA insertion in number (3-9, 4-10, respectively). Type III has a TAAATG repeated two times. The GenBank accession numbers of them are as follows: GQ370904-GQ370934.

**Figure 5:**
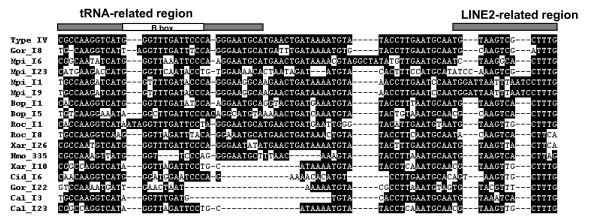
**Alignment of HAmo Type IV members**. The consensus sequences are shown on top. The most frequently occurring nucleotide at a position was chosen as the consensus nucleotide at the position. The tRNA-derived region and LINE-related region of SINE are shown by thick bar. The GenBank accession numbers of them are as follows: GQ370935-GQ370949.

**Figure 6:**
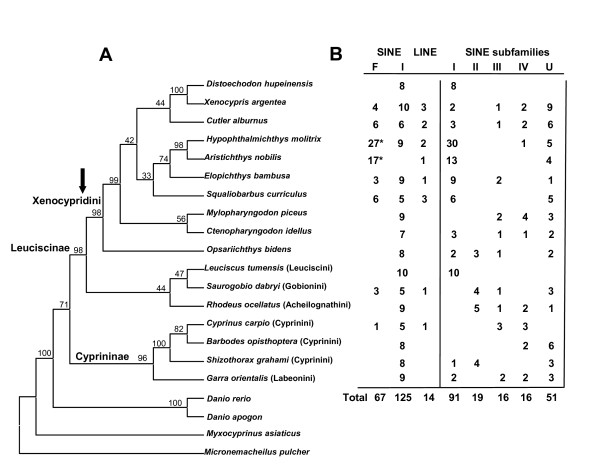
**Mapping different subfamilies and copy numbers to the phylogenetic tree**. **A) **Based on the rag2 sequences, the phylogenetic tree of 17 fishes representing different tribes of Cyprinidae family was constructed using Bayesian inference. Among them, the rag2 sequences of three species *Distoechodon hupeinensis*, *Shizothorax grahami*, *Barbodes opisthoptera *was substituted by another closely related species in the same genus respectively, because the absence of their rag2 sequences in GenBank. The trees resulting from phylogenetic analyses of the RAG2 gene place the Danioninae as the basal-most group in the family and divide the remaining cyprinids into two divergent subfamilies Cyprininae and Leuciscinae. The arrow denoted the Xenocyprinidini clade, containing silver carp, bighead carp and their closely related species endemic to East Asia. **B) **This part listed the clone numbers of SINE and LINE isolated from each species and the copy numbers estimated by qRT-PCR. The mapping results shows the four subfamilies, as multiple active source genes, expanded preferentially in a certain lineage or the whole family of Cyprinidae. The star indicated that the whole clones contain 21 and 15 of HAmo type I reported previously. The seventh column (U) denoted the unidentified SINE sequences that cannot be classified into a kind of type.

Figure 3, Figure 4 and Figure 5 show the alignments of the four subfamilies. Type I is supported by 91 sequences from 12 species, with a calculated average sequence divergence of 3.7%. It contains 34 of our previously reported young HAmo sequences from silver carp and bighead carp [1], which have been identified as having proliferate recently. Type II and III are divided by only five dispersed diagnostic nucleotides and they expanded preferentially in the different lineages (Figure 6, see discussion). Type IV may be the most divergent subfamily because many members have different short deletion regions. We deduced that type I may be the youngest subfamily based on the small divergence among its members and on the expansion that is evident in some species (the Xenocyprinidini clade), which likely was generated very recently on the time scale. Moreover, most of the type I sequences in silver carp and bighead carp are species-specific and even not fixed among the population [1].

The four subfamilies can be distinguished from each other mainly by their different tRNA-unrelated regions. The different patterns of insertion and duplication in different subfamilies constitute the most diagnostic nucleotides that lead to division of the family into different subfamilies. Type I has a variable polyA (1-14) insertions and a TATAA repeat two to three times. Type II has two variable polyA(3-9, 4-10) insertions whereas type III has a TAAATG sequence that repeats two times.

### The common tail is conserved in primary and secondary structures between HAmo SINE and HAmo LINE

Many HAmo LINE2 clones were obtained simultaneously when isolating HAmo SINEs (Figure 6) because the probe contained a tail region shared by HAmo SINE and its partner LINE. A consensus sequence of the HAmo LINE2 tail was constructed by aligning all 14 isolated HAmo LINEs (in this paper) plus 14 previously reported in silver carp and bighead carp. The consensus sequences of the four HAmo SINE subfamilies shared an almost identical 42-bp-long 3'tail to HAmo LINE (Figure 2), suggesting that HAmo SINE may borrow the enzymatic machinery of HAmo LINE to proliferate in the cyprinid genomes through the conserved 3' -tail.

### Estimation of copy number using quantitative RT-PCR

To estimate the number of copies of HAmo SINE in each species, the pair of primers used in internal-primer-SINE-PCR was also used to amplify the HAmo SINE sequences in genomic DNA (sample) and in the plasmid Hmo41_It (standard) using RT-PCR. We used two diluted genomic DNA as tested samples to perform PCR reaction. The final estimations of copy numbers using different concentrations of the genomic DNA template were very close, suggesting that the results of the experiment were stable and efficient. Table 2 summarizes the detailed results. The copy numbers of HAmo elements in 12 cyprinid genomes were estimated to vary from 10^4 ^to 10^6^. In other words, HAmo SINE may constitute 0.1-10% of cyprinid genomes (about 10^9 ^bp). Considering the possibility of mismatch between primers with more divergent HAmo SINE sequences, the results of quantitative RT-PCR were minimal estimates of HAmo SINE copy numbers in respective genomes.

### HAmo SINE in zebrafish genome

Danio rerio was a cyprinid fish belonging to Danioninae, Cyprinidae. In previous research, the trees resulting from cyprinid phylogenetic analyses of the RAG2 gene place the Danioninae as the basal-most group in the family and divide the remaining cyprinids into two divergent subfamilies Cyprininae and Leuciscinae [19]. In this paper, 17 cyprinid fish were selected for representing various taxa within Cyprinidae, and their phylogeny based on rag2 gene (Figure 6) also revealed the pattern that within the Cyprinidae, Danio emerged at first and the remaining cyprinid species clustered into two major clades, Cyprininae and Leuciscinae. The basal relationship of the Danioninae, relative to other Cyprinidae, has also been confirmed in other recent molecular analyses [20,21].

For investigating the HAmo SINE in zebrafish, the consensus sequences of four subfamilies (type I, II, III and IV) were used as queries to blastn Danio rerio database in NCBI website. Because the primer regions of these consensus sequences are identical and uninformative, we eliminated these regions from the whole consensus sequences, as shown in Figure 2. These consensus sequences were about 83-91 bp in length containing about 31-37 bp central region and partial tRNA-related region and LINE2-derived region. We set the parameter e = 0.1 and count the number of similar sequences with query (coverage > 90%) in zebrafish genome. 16 sequences (identity 72%-78%), 40 sequences (identity 72%-81%), 58 sequences (identity 72%-80%, and other 2 sequence have high similarity: 1 with 85%, 1 with 87%) were found to be similar with the consensus sequences of type I, II and III, respectively. Because these similar sequences in zebrafish are too divergent with the corresponding type I, II and III consensus sequences, especially in the central region representing the most specific diagnostic nucleotide, we couldn't determine the presence of type I, II and III of HAmo SINE in zebrafish. As our described, Type I mainly distributed in lineage Leuciscinae and Type II were found only in some species. Their restricted species distribution may lead to the absence of them in primitive cyprinid species Danio rerio. In contrast, we found 105 sequences similar with consensus sequence of type IV with identity 72%-90% in zebrafish. Majority of them can be identified and characterized to be members of type IV since type IV were the oldest subfamily and its members have more divergence between sequences and dispersed in the whole Cyprinidae. The alignment of type IV sequences in zebrafish with the consensus sequence of type IV in this paper was provided as Additional file 1.

Moreover, among our isolated HAmo internal sequences in 17 cyprinid fishes, there are tens of HAmo sequences that have more divergent central region with each other as well as with the type I, II, III and IV (Figure 6, type U, Additional file 2). We couldn't divide them into specific subfamilies now because of lacking enough supported sequences. When using them as queries to blastn Danio rerio genome, tens or hundreds sequences similar with many queries have been found in zebrafish. In addition, we also obtained 7 HAmo SINE sequences by cloning the inter-primer SINE PCR products in zebrafish, provided in Additional file 3. All these evidences manifest that numerous HAmo SINE copies presented in the primitive cyprinid fish Danio rerio and dispersed in the whole Cyprinidae.

## Discussion

### Multiple source genes are responsible for amplification of the HAmo SINE in Cyprinid genomes

In this work, we isolated and characterized many HAmo SINEs from 17 species of the Cyprinidae family. The results show that the HAmo SINE is widespread in cyprinid genomes and that copy numbers are about 10^4^-10^6^.

Based on the rag2 gene, we constructed the phylogenetic relationships among the 17 species studied in order to map the information obtained from isolated clones in each species (Figure 6). The ratio of different subfamily clones found in each species could roughly reflect the lineage in which the subfamilies expanded preferentially, although potential statistical bias resulting from the small number of clones analyzed exists. The result of this analysis showed that type I preferentially expanded in the clade Leuciscinae, especially in the tribe Xenocyprinidini, which contained many young and closely related endemic taxa from East Asia. In contrast, Type II distributed on some non- Xenocyprinidini species, whereas Type III and IV seemed to be dispersed throughout the whole Cyprinidae family. Judging from the distribution of members of the same sequence subfamily of SINEs within different lineages and from the distribution of the different-sequence subfamilies within the same lineage, we concluded that multiple dispersed loci are responsible for the amplification of SINEs in cyprinid genomes. In other words, multiple rivalrous source genes (subfamilies) expanded in cyprinid genomes and showed different advantages of expansion in different lineages. The different advantage of expansion among subfamilies may results from the retropositional regulation to source genes in the process of transcription and selection at the RNA level. Many factors, such as the chromatin context near a newly transposed SINE, methylation, cis-acfing promoter elements, and trans-acting factors involve in this process [22-24]. Because the effects of these factors might differ among species, frequencies of retroposition might also differ among specific lineages [25].

From the mapping results, type I seems to have expanded actively in the Leuciscinae clade and to have undergone retroposition among populations of silver carp and bighead carp. However, type I seems not to have flourished in the Cyprininae clade, as there are only two clones found in two species. Thus, we deduced that the master (source) gene of type I was first generated in a common ancestor of the family Cyprinidae, and then this gene was vertically inherited during evolution of the cyprinid lineage. A highly dominant source gene for the type I subfamily may have been newly created by retroposition and actively amplified in the Leuciscinae clade. This scenario indicates that the actual sequence of this subfamily might not be the cause of the high retropositional efficiency of SINEs. A local environment such as the site of retroposition, RNA secondary structure, and promoter activity might be crucial for the establishment of a highly dominant source gene during evolution [25].

The above analysis indicates that multiple source genes (subfamilies) actively amplified within a certain lineage or within the whole family of Cyprinidae. Therefore, the changes (diagnostic nucleotides) that occurred in the tRNA-unrelated region may not affect the efficiency of retroposition of different actively dominant source genes (subfamilies). The high efficiency and successful proliferation of HAmo SINEs might have occurred for two reasons. First, HAmo SINEs retain the overall secondary structure and conserved the A and B box in the tRNA-related region, which ensures the RNA pol III recognition and transcriptional activity of SINEs. Second, the 3' tail of HAmo SINEs and HAmo LINE2 is almost identical in primary sequence and secondary structures, which allows the former to utilize the LINE2 enzymatic machinery.

### The species distribution of HAmo SINE

**Figure 7:**
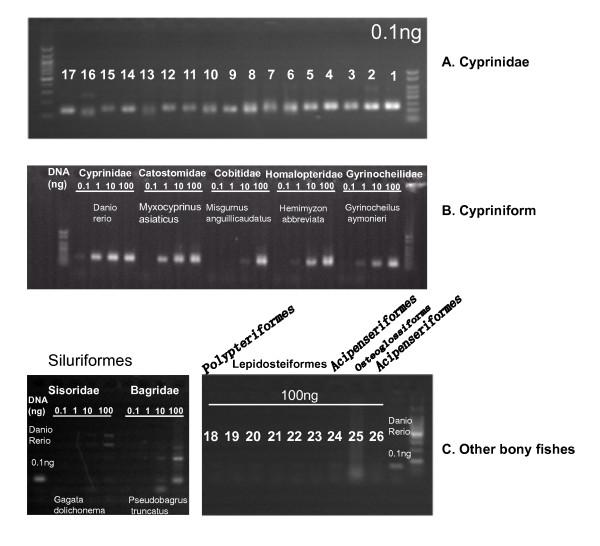
**Internal-primer SINE PCR was conducted to examine the species distribution of HAmo SINE**. The PCR was run in a total volume of 25 μl with 25 cycles of 95°C for 40s, 58°C for 40s, and 72°C for 40s. Four ten-fold DNA: 0.1ng, 1ng, 10ng, 100ng were used as template for each species. The expected fragment was about 130 bp. A) The investigated species from Cyprinidae in this paper. From number 1-17: 1. *Hypophthalmichthys molitrix*; 2. *Aristichthys nobilis*; 3. *Distoechodon hupeinensis*; 4. *Xenocypris argentea*; 5. *Cutler alburnus*; 6. *Leuciscus tumensis*; 7. *Ctenopharyngodon idellus*; 8. *Mylopharyngodon piceus*; 9. *Squaliobarbus curriculus*; 10. *Elopichthys bambusa*; 11. *Rhodeus ocellatus*; 12. *Saurogobio dabryi*; 13. *Cyprinus carpio*; 14. *Shizothorax grahami*; 15. *Barbodes opisthoptera*; 16. *Opsariichthys bidens*; 17. * Garra orientalis*. B) Four species were used for representing four families of order Cypriniforms: *Myxocyprinus asiaticus *(Catostomidae), *Misgurnus anguillicaudatus *(Cobitidae), *Gyrinocheilus aymonieri *(Gyrinocheilidae) and *Hemimyzon abbreviata *(Homalopteridae). C) In Siluriformes, two species were used: Gagata dolichonema (Sisoridae), Pseudobagrus truncatus (Bagridae). Other orders:18. *Polypterus delhezi *(Polypteriformes); 19. *Lepisosteus osseus *(Lepidosteiformes); 20. *Atractosteus tropicus *(Lepidosteiformes); 21. *Atractosteus spatula *(Lepidosteiformes); 22. *Lepisosteus platyrhincus *(Lepidosteiformes); 23. *Lepisosteus platostomus *(Lepidosteiformes). 24. *Polyodon spathala *(Acipenseriformes); 25. Osteoglossiforms; 26. *Acipenser sinensis *(Acipenseriformes).

To determine the species distribution of HAmo SINE, Inter-primer SINE PCR was also conducted in non-cyprinid fishes. Four different ten-fold DNA dosage: 0.1 ng, 1 ng, 10 ng, 100 ng were used for each species. Firstly, for 17 cyprinid fishes, visible PCR band could be observed when 0.1 ng DNA was used as template in PCR (Figure 7A). Secondly, in the case of DNA of non-cyprinid fishes in Cypriniform, PCR products were observed when 1 ng DNA of Myxocyprinus asiaticus (Catostomidae), Hemimyzon abbreviata (Homalopteridae), Gyrinocheilus aymonieri (Gyrinocheilidae) and 10 ng DNA of Misgurnus anguillicaudatus (Cobitidae) were used, as shown in Figure 7B. We cloned and sequenced these PCR products from these species. 3-7 HAmo SINE related sequences with average identity about 70% were obtained in each species, see Additional file 3. All these sequences and the gel electrophoresis show that HAmo SINE-related sequences may presented in the other families of order Cypriniform. We deduced that the more DNA (1 ng or 10 ng compared to 0.1 ng) must be used in PCR for detection of HAmo SINE may result from the mutation in primer-matched regions of HAmo sequences in the investigated species since the investigated species diverged more with cyprinids. More evidence and sequences need to be obtained to determine the SINE family in other families of order Cypriniform.

In Siluriformes, two species were used: Gagata dolichonema (Sisoridae), Pseudobagrus truncatus (Bagridae). Some PCR products were generated only when 100 ng DNA template were used (Figure 7C). We sequenced the PCR products and found that they belong to the non-specific amplified products. In the case of other order of bony fishes, the PCR product corresponding to the HAmo SINE elements was not detected when 100 ng DNA was used for each sample of the order Polypteriformes, Lepidosteiformes, Acipenseriformes, Osteoglossiforms.

Thus, the PCR analysis has demonstrated that the genomes of representatives of families Catostomidae, Cobitidae, Gyrinocheilidae and Homalopteridae may contain HAmo related SINE elements whereas the genomes of other order Siluriformes, Polypteriformes, Lepidosteiformes, Acipenseriformes, Osteoglossiforms may do not carry this retroposon.

## Conclusions

In conclusion, by searching for HAmo SINEs from different taxa within the Cyprinidae, we found that HAmo SINEs were widespread in cyprinid genomes. The hundreds of SINE sequences found in our study revealed that multiple source subfamilies actively expanded and underwent retroposition within a certain lineage or within the whole family of Cyprinidae. Thus, HAmo SINE should be a useful phylogenetic marker for future analyses of the evolutionary relationships among species in the Cyprinidae family.

## Abbreviations

SINE: short interspersed repetitive elements; LINE: long interspersed repetitive elements; qRT-PCR: Quantitative Real Time-PCR; RT: reverse transcriptase.

## Authors' contributions

SH and CT conceived and designed the experiments, CT and XG performed the experiments and analyzed the data, CT and SH wrote the paper. All authors read and approved the final manuscript.

## Supplementary Material

Additional file 1 **Type IV of HAmo SINE in zebrafish**. The alignment of type IV sequences in zebrafish with the consensus sequence of type IV in this paper was provided. Click here for file

Additional file 2 **Type U of HAmo SINE in cyprinid fishes**. Among our isolated HAmo internal sequences in 17 cyprinid fishes, there are tens of HAmo sequences that have more divergent central region with each other as well as with the type I, II, III and IV. We defined them as type U (unidentified) because we couldn't divide them into specific subfamilies now. Click here for file

Additional file 3 **HAmo SINE in zebrafish and other families of order Cypriniform isolated from inter-primer SINE PCR products**. HAmo SINE and HAmo SINE-related sequences by cloning the inter-primer SINE PCR products were isolated in zebrafish and some species from families of order Cypriniform. Dre stands for Danio rerio(Cyprinidae); Gay stands for Gyrinocheilus aymonieri (Gyrinocheilidae); Hab stands for Hemimyzon abbreviata (Homalopteridae); Mas stands for Myxocyprinus asiaticus (Catostomidae). Click here for file
